# Surgical outcomes of uveitic glaucoma

**DOI:** 10.1007/s12348-010-0012-8

**Published:** 2010-11-18

**Authors:** Ester Carreño, Sonia Villarón, Alejandro Portero, José M. Herreras, José A. Maquet, Margarita Calonge

**Affiliations:** 1 Ocular Immunology Unit-IOBA (Instituto Universitario de Oftalmobiología), University of Valladolid, Campus Miguel Delibes, Camino del Cementerio s/n, 47011 Valladolid, Spain; 2 Hospital Clínico Universitario de Valladolid, Av. Ramón y Cajal s/n, 47005 Valladolid, Spain

**Keywords:** Glaucoma, Glaucoma surgery, Mitomycin C, Trabeculectomy, Uveitis

## Abstract

**Purpose:**

Secondary glaucoma is a difficult and frequent complication of uveitis. The aim of this study is to describe the results of surgery in uveitic glaucoma and to identify situations linked to a high risk of failure of the surgery.

**Methods:**

Retrospective observational study. Clinical and surgical data from 27 cases of uveitic glaucoma that underwent glaucoma surgery over a period of 9 years were collected.

**Results:**

The main diagnosis and aetiology were anterior uveitis (61.90%) and herpes (38.10%) respectively. Trabeculectomy with mitomycin C was performed in 51.9% of the cases. An intraocular pressure lower than 16 mmHg and managed with less than two drugs was achieved in 48.15% of the cases. Higher risks of surgical failure were associated with intermediate uveitis, idiopathic uveitis, Fuchs’ cyclitis, combined surgery with phacoemulsification, omission of mitomycin C, intraocular inflammation at surgery and relapse of the uveitis.

**Conclusions:**

There are some situations linked to a high risk of failure of surgery in uveitic glaucoma, which should be avoided when possible, mainly the association of higher risk with combined approaches.

## Introduction

Secondary uveitic glaucoma is a common complication of intraocular inflammation and is present in up to 20% of patients with uveitis [1, 2]. Several mechanisms are involved in the pathogenesis of uveitic glaucoma [1]. Uveitic glaucoma often gets worse despite intensive medical treatment, and it may require surgical intervention. Surgical management is challenging because of the increased risk of post-operative inflammation and failure to control intraocular pressure (IOP) [3]. The aim of this study was to analyse the different variables that play a role in the final surgical outcome of uveitic glaucoma in a cohort of patients with intraocular inflammatory disease attending a referral centre.

## Materials and methods

All patients with uveitic glaucoma who underwent glaucoma surgery at the Hospital Clínico Universitario de Valladolid over a period of 9 years (1999–2007) were reviewed. All procedures conformed to the tenets of the Declaration of Helsinki, and the study was approved by the Institutional Review Board of that hospital. Data collected from clinical charts included age, sex, affected eye, predominant anatomic location of inflammation, aetiology, course, time of follow-up, time between the diagnosis of uveitis and the diagnosis of glaucoma, time between the diagnosis of glaucoma and the surgery, state of the angle at time of surgery, pre- and post-surgical visual acuity and IOP, uveitic activity at time of surgery, type of surgery, use of anti-metabolites, flare-up of uveitis after surgery, complications, necessity for a second surgery, final outcome, and where relevant, time to the failure. A case was considered as one surgical procedure for glaucoma; thus the same eye could be considered as two separate cases if re-intervention was necessary.

Uveitis was diagnosed based on clinical examination. Types of uveitis, anatomic classification, and course were defined according to the criteria proposed by the International Uveitis Study Group [4] and by aetiology [5]. Patients were considered to have uveitic glaucoma if there was no previous history of glaucoma or IOP higher than 21 mmHg prior to the onset of uveitis. Furthermore, after diagnosis of uveitis, the patients developed an IOP higher than 21 mmHg that required anti-glaucoma treatment and optic disc abnormalities characteristic of glaucoma. For the patients in this study, the IOP was not controlled by topical medication and/or there was evidence of deterioration of glaucoma defined by worsening of the visual field, indicating the need for surgery.

One week prior to surgery, all the patients got 40 mg of transeptal triamcinolone. Beginning 3 days before surgery, patients were given dexamethasone eyedrops every 2 h to prevent flare-up. If required, basal treatment of the uveitis was maintained. Post-surgical treatment consisted of a commercial combination of dexamethasone and antibiotic (tobramicine or neomicine sulfate plus polimixine-B-sulfate) eye drops every 2 h for the first week with tapering thereafter as needed.

Surgical procedures included trabeculectomy, phacotrabeculectomy, and Ahmed valve implantation. For each type of surgery, some cases were treated with the anti-metabolite mitomycin C (MMC). The concentration of MMC was determined according to the risk of the surgery (Table 1), the cases were MMC was not used are previous cases to the application of this protocol [6]. A 3 × 5 mm piece of sponge (Espongostan Film, Ferrosan A/S, Copenhagen, Denmark) soaked with MMC was placed under the conjunctival flap over the scleral bed in trabeculectomy and over the plate in sub-Tenon space in Ahmed valve for 2 min. Subsequently, the sponge was removed and it was copiously irrigated with balanced salt solution. Trabeculectomy was performed with the limbus-base technique, and phacoemulsification was achieved with a clear corneal incision and intraocular lens implantation. All surgeries were executed by the same surgeon (J.A.M.) and most occurred after at least 6 months of inflammation control. In a few cases, it was imperative to control the IOP even though the intraocular inflammation was not fully under control. Follow-up visits were held on days 1–3 post-surgery, then weekly until 1 month after the surgery. Visits were then held monthly until 3 months after surgery and then every 3 months until 1 year after surgery. After that, follow-up visits were held every 6 months unless complications arose.

**Table 1:** Protocol to select MMC concentration

Risk factor	Assigned risk	MMC concentration (mg/ml)
Previous surgery (conjunctiva)	6	
Combined surgery	5	
Secondary glaucoma	4	
Target IOP < 16 mmHg	4	
Topical treatment >1 year	3	
Age < 40 years	3	
Diabetes melitus	1	
Previous laser trabeculoplasty	1	
	<2	No MMC
	3–5	0.1
	6–9	0.2
	>10	0.4

Outcomes were measured by changes in visual acuity and IOP with or without anti-glaucoma medication. Changes in visual acuity were defined as increases or decreases of ≥1 line in Snellen charts from the initial best-corrected visual acuity. IOP was measured with a Goldman applanation tonometer.

The final outcome after surgery was classified as: complete success (IOP ≤16 mmHg with no drugs or only one drug for control), partial success (IOP ≤16 mmHg with ≥2 drugs for control) and failure (IOP >16 mmHg). The value of IOP considered for final outcome was the value recorded at the patient’s last visit during the study period.

All the data were collected in an Access database (Microsoft Office Access 2007, Microsoft Corporation, Redmond, WA, USA) and further analysed by SPSS (SPSS version 15.0, SPSS Inc., Chicago, IL, USA). Descriptive analyses were performed, and statistics were presented as means ± standard deviations and as 99.61% confidence intervals (CI 99.61%), according with Bonferroni correction for an α = 0.05.

## Results

Data were collected from 19 patients with a total of 21 eyes. Including surgeries on the same eye, there were 27 cases (in two cases, one re-surgery and in two cases, two re-surgeries, Table 2 resume the cases where a re-intervention was needed). There were no significant differences in gender (11 males; eight females) and laterality (13 right eyes; 14 left eyes). The mean age was 48.3 ± 15.0 years (mean ± SD), and the mean follow-up period was 69.6 ± 54.9 months (range, 8–204 months). Age was not a factor in the final outcome and visual acuity (Table 3). Most of the patients (61.90%) had anterior uveitis (Table 4). Eight of the uveitis were due to herpes infection (38.10%), followed by Fuchs’ cyclitis (14.29%) and ankylosing spondylitis (14.29%) (Table 4).

**Table 2:** Procedures and final outcome of patients who needed re-surgery

Case	First procedure	Second procedure	Third procedure	Final outcome
1	Trabeculectomy + MMC	Ahmed valve + MMC		Partial success
2	Trabeculectomy + MMC	Ahmed valve		Total success
3	Phacotrabeculectomy + MMC	Trabeculectomy + MMC	Ahmed valve	Total success
4	Phacotrabeculectomy	Trabeculectomy + MMC	Ahmed valve	Failure

**Table 3:** Relationship between final outcome, visual acuity and age

	*n*/27 (%)	Age^a^
Final outcome		
Complete success	13/27 (48%)	49.77 ± 16.50
Partial success	3/27 (11%)	61.67 ± 17.04
Failure	11/27 (41%)	42.40 ± 9.69
Visual acuity		
Improved	15/27 (56%)	48.73 ± 14.77
Unchanged	5/27 (18%)	48.50 ± 19.96
Worsened	7/27 (26%)	47.28 ± 14.97

^a^Mean ± standard deviation

**Table 4:** Anatomic classification, aetiology and course of uveitic disease

	Number	Per cent
Anatomic classification		
Anterior uveitis	13	62
Intermediate uveitis	5	24
Panuveitis	3	14
Uveitis aetiology		
Herpes	8	38
Fuchs’ heterochromic cyclitis	3	14
Ankylosing spondylitis	3	14
Tuberculosis	2	10
Anterior idiophatic	1	5
Behçet’s disease	1	5
Pars planitis	1	5
Posner–Schlossman syndrome	1	5
Sarcoidosis	1	5
Course of uveitis		
Recurrent	13	62
Chronic	8	38

Twenty-two cases (81.5%) took >1 year from the diagnosis of the uveitis to glaucoma. For 21 cases, >1 year elapsed between the diagnosis of the glaucoma and surgery. Twenty-three cases (85.2%) had open angles before surgery, and two (7.4%) had closed angles. In one case, the angle was open with synechiaes, and in another case there was an iris plateau. In two cases, elevated IOP necessitated glaucoma surgery before inflammation was under control.

Trabeculectomy with MMC was performed in 14 cases (51.9%) while six cases had combined phacoemulsification and trabeculectomy (five with MMC (18.5%) and one without MMC (3.7%)) and seven cases had Ahmed valve insertions (six without MMC (22.2%), and one with MMC (3.7%)). Re-intervention was needed in 12 cases (44.4%), with no statistically significant relationship to either anatomic classification or aetiology.

Complete success was achieved in 13 cases (48.2%), partial success in three cases (11.1%), and failure occurred in 11 cases (40.7%). The mean time from surgery to failure was 47.5 ± 58.7 months (range, 4–192 months). Most failures (36.4%) occurred during the first year of follow-up while 27.3% occurred during the second year and 36.36% after >5 years of follow-up.

The pre-surgical IOP was 31.9 ± 9.9 mmHg (CI 95%, 28.0–35.7 mmHg), and the post-surgical IOP for all cases at the end of the follow-up period was 17.3 ± 9.1 mmHg (CI 95%, 13.8–20.8). The visual acuity increased after surgery in 15 cases (55.6%) and decreased in seven cases (25.9%). It remained stable in five cases (18.5%). There was no correlation between outcomes of visual acuity and IOP.

In most cases, there was also no correlation in the final IOP outcome or visual acuity according with the anatomic classification (Table 5). The exception was for panuveitis where all three cases were total successes with regard to IOP management, and two of them had improved visual acuity. With regard to aetiology, the final IOP outcome was worst for anterior idiopathic uveitis, where all cases failed, and for Fuchs’ cyclitis, where most of the cases failed (Table 6), although these differences were not statistically significant in the second case. However, in relation with the aetiology and course of the uveitis, there were neither significant differences in the final visual acuity nor IOP outcome (Table 7).

**Table 5:** Final IOP outcome and visual acuity according to anatomic classification

	Final IOP outcome						Visual acuity					
	Complete success		Partial success		Failure		Improvement		Unchanged		Worsening	
	*n* (%)	IC (99.61%)	*n* (%)	IC (99.61%)	*n* (%)	IC (99.61%)	*n* (%)	IC (99.61%)	*n* (%)	IC (99.61%)	*n* (%)	IC (99.61%)
Anterior uveitis	8 (44)	10–78%	3 (17)	0–42%	7 (39)	6–72%	10 (56)	22–90%	3 (17)	0–42%	5 (28)	0–58%
Intermediate uveitis	2 (33)	0–89%	0 (0)	–	4 (67)	11–100%	3 (50)	0–100%	2 (33)	0–89%	1 (17)	0–61%
Posterior uveitis	0 (0)	–	0 (0)	–	0 (0)	–	0 (0)	–	0 (0)	–	0 (0)	–
Panuveitis	3 (100)	–	0 (0)	–	0 (0)	–	2 (67)	0–100%	0 (0)	–	1 (33)	0–100%

**Table 6:** Final IOP outcome and visual acuity according to aetiology of the uveitis

	Final IOP outcome						Visual acuity					
	Complete success		Partial success		Failure		Improvement		Unchanged		Worsening	
	*n* (%)	IC (99.61%)	*n* (%)	IC (99.61%)	*n* (%)	IC (99.61%)	*n* (%)	IC (99.61%)	*n* (%)	IC (99.61%)	*n* (%)	IC (99.61%)
Herpes	6 (67)	22–100%	1 (11)	0–41%	2 (22)	0–62%	6 (67)	22–100%	1 (11)	0–41%	2 (22)	0–62%
Fuchs’ heterochromic cyclitis	1 (20)	0–72%	0 (0)	–	4 (80)	28–100%	3 (60)	0–100%	1 (20)	0–72%	1 (20)	0–72%
Ankylosing spondylitis	2 (50)	0–100%	0 (0)	–	2 (50)	0–100%	1 (25)	0–87%	1 (25)	0–87%	2 (50)	0–100%
Anterior idiophatic	0 (0)	–	0 (0)	–	3 (100)	–	2 (67)	0–100%	0 (0)	–	1 (33)	0–100%
Tuberculosis	0 (0)	–	2 (100)	–	0 (0)	–	2 (100)	–	0 (0)	–	0 (0)	–
Behçet disease	1 (100)	–	0 (0)	–	0 (0)	–	0 (0)	–	0 (0)	–	1 (100)	–
Pars planitis	1 (100)	–	0 (0)	–	0 (0)	–	0 (0)	–	1 (100)	–	0 (0)	–
Posner–Schlossman	1 (100)	–	0 (0)	–	0 (0)	–	0 (0)	–	1 (100)	–	0 (0)	–
Sarcoidosis	1 (100)	–	0 (0)	–	0 (0)	–	1 (100)	–	0 (0)	–	0 (0)	–

**Table 7:** Final IOP outcome and visual acuity according to course of uveitic disease

	Final IOP outcome						Visual acuity					
	Complete success		Partial success		Failure		Improvement		Unchanged		Worsening	
	*n* (%)	IC (99.61%)	*n* (%)	IC (99.61%)	*n* (%)	IC (99.61%)	*n* (%)	IC (99.61%)	*n* (%)	IC (99.61%)	*n* (%)	IC (99.61%)
Acute	9 (53)	18–88%	1(6)	0–22%	7 (41)	7–75%	9 (53)	18–88%	3 (18)	0–45%	5 (29)	0–61%
Chronic	4 (40)	0–84%	2 (20)	0–56%	4 (40)	0–84%	6 (60)	15–100%	2 (20)	0–56%	2 (20)	0–56%

There was no statically difference in success or visual acuity according to the surgical procedure (Table 8), to the interval between the diagnosis of uveitis and glaucoma or the time between the diagnosis of glaucoma and surgery (Tables 9 and 10).

**Table 8:** Final IOP outcome and visual acuity according to surgical procedure

	Final IOP outcome						Visual acuity					
	Complete success		Partial success		Failure		Improvement		Unchanged		Worsening	
	*n* (%)	IC (99.61%)	*n* (%)	IC (99.61%)	*n* (%)	IC (99.61%)	*n* (%)	IC (99.61%)	*n* (%)	IC (99.61%)	*n* (%)	IC (99.61%)
Trabeculectomy with MMC	9 (64)	27–100%	0 (0)	–	5 (36)	0–73%	7 (50)	12–88%	4 (29)	0–64%	3 (21)	0–53%
Trabeculectomy without MMC	0 (0)	–	0 (0)	–	0 (0)	–	0 (0)	–	0 (0)	–	0 (0)	–
Combined with MMC	1 (20)	0–72%	2 (40)	0–100%	2 (40)	0–100%	4 (80)	28–100%	1 (20)	0–72%	0 (0)	–
Combined without MMC	0 (0)	–	0 (0)	–	1 (100)	–	1 (100)	–	0 (0)	–	0 (0)	–
Ahmed valve with MMC	0 (0)	–	1 (100)	–	0 (0)	–	1 (100)	–	0 (0)	–	0 (0)	–
Ahmed valve without MMC	3 (50)	0–100%	0 (0)	–	3 (50)	0–100%	2 (33)	0–88%	0 (0)	–	4 (67)	12–100%

**Table 9:** Final IOP outcome and visual acuity according to time to diagnosis of glaucoma

	Final IOP outcome						Visual acuity					
	Complete success		Partial success		Failure		Improvement		Unchanged		Worsening	
	*n* (%)	IC (99.61%)	*n* (%)	IC (99.61%)	*n* (%)	IC (99.61%)	*n* (%)	IC (99.61%)	*n* (%)	IC (99.61%)	*n* (%)	IC (99.61%)
>1 year for glaucoma	10 (45)	15–75%	3 (14)	0–35%	9 (41)	11–71%	12 (55)	24–86%	4 (18)	0–42%	6 (27)	0–54%
<1 year for glaucoma	2 (50)	0–100%	0 (0)	–	2 (50)	0–100%	3 (75)	13–100%	0 (0)	–	1 (25)	0–87%

**Table 10:** Final IOP outcome and visual acuity according to time to surgery from diagnosis of glaucoma

	Final IOP outcome						Visual acuity					
	Complete success		Partial success		Failure		Improvement		Unchanged		Worsening	
	*n* (%)	IC (99.61%)	*n* (%)	IC (99.61%)	*n* (%)	IC (99.61%)	*n* (%)	IC (99.61%)	*n* (%)	IC (99.61%)	*n* (%)	IC (99.61%)
>1 year for surgery	10 (48)	17–79%	3 (14)	0–36%	8 (38)	8–68%	12 (57)	26–88%	3 (14)	0–36%	6 (29)	1–57%
<1 year for surgery	3 (50)	0–100%	0 (0)	–	3 (50)	0–100%	3 (50)	0–100%	2 (33)	0–88%	1 (17)	0–61%

The interval between diagnosis of uveitis and glaucoma was significantly correlated with the interval between diagnosis of glaucoma and surgery (Fisher’s exact test, *p* = 0.028). Thus, longer times before the onset of glaucoma were associated with longer times to surgery.

All cases of active uveitis at the time of surgery failed. In contrast just 36% of cases of inactive uveitis at the time of surgery failed. Similarly, the visual acuity worsened in 50% of active uveitis, but just in 24% of inactive uveitis (Table 11).

**Table 11:** Final IOP outcome and visual acuity according to the inflammatory activity of the uveitis at time of surgery

	Final IOP outcome						Visual acuity					
	Complete success		Partial success		Failure		Improvement		Unchanged		Worsening	
	*n* (%)	IC (99.61%)	*n* (%)	IC (99.61%)	*n* (%)	IC (99.61%)	*n* (%)	IC (99.61%)	*n* (%)	IC (99.61%)	*n* (%)	IC (99.61%)
Active uveitis at surgery	0 (0)	–	0 (0)	–	2 (100)	–	1 (50)	0–100%	0 (0)	–	1 (50)	0–100%
Inactive uveitis at surgery	13 (52)	23–81%	3 (12)	0–31%	9 (36)	8–64%	14 (56)	27–85%	5 (20)	0–43%	6 (24)	0–49%

Relapse of the uveitis increases the risk of failure of the surgery, although the differences were not statistically significant (Table 12).

**Table 12:** Final IOP outcome and visual acuity according to the relapse of the uveitis

	Final IOP outcome						Visual acuity					
	Complete success		Partial success		Failure		Improvement		Unchanged		Worsening	
	*n* (%)	IC (99.61%)	*n* (%)	IC (99.61%)	*n* (%)	IC (99.61%)	*n* (%)	IC (99.61%)	*n* (%)	IC (99.61%)	*n* (%)	IC (99.61%)
Relapse of uveitis	1 (20)	0–72%	0 (0)	–	4 (80)	28–100%	5 (100)	–	0 (0)	–	0 (0)	–
No relapse of uveitis	12 (55)	25–85%	3 (14)	0–35%	7 (32)	9–55%	10 (45)	16–74%	5 (23)	9–37%	7 (32)	9–55%

Treatment with MMC had a mild positive effect on the rate of complete success and improved visual acuity, but without a statistical significant difference (Table 13).

**Table 13:** Final IOP outcome and visual acuity according to the use of anti-metabolites (mitomycin C (MMC))

	Final IOP outcome						Visual acuity					
	Complete success		Partial success		Failure		Improvement		Unchanged		Worsening	
	*n* (%)	IC (99.61%)	*n* (%)	IC (99.61%)	*n* (%)	IC (99.61%)	*n* (%)	IC (99.61%)	*n* (%)	IC (99.61%)	*n* (%)	IC (99.61%)
MMC	10 (50)	18–82%	3 (15)	0–41%	7 (48)	17–79%	12 (60)	28–92%	5 (25)	0–53%	3 (15)	0–41%
No MMC	3 (43)	0–97%	0 (0)	–	4 (57)	3–100%	3 (43)	0–97%	0 (0)	–	4 (57)	3–100%

The combination of glaucoma and cataract surgery had a negative effect on the complete success rate, but not in visual acuity, although there were not statistical significant differences (Table 14).

**Table 14:** Final IOP outcome and visual acuity according to the combination of Phacoemulsiphication to glaucoma surgery

	Final IOP outcome						Visual acuity					
	Complete success		Partial success		Failure		Improvement		Unchanged		Worsening	
	*n* (%)	IC (99.61%)	*n* (%)	IC (99.61%)	*n* (%)	IC (99.61%)	*n* (%)	IC (99.61%)	*n* (%)	IC (99.61%)	*n* (%)	IC (99.61%)
Phacoemulsification	1 (17)	0–61%	2 (33)	0–88%	3 (50)	0–100%	5 (83)	39–100%	1 (17)	0–61%	0 (0)	–
No phacoemulsification	12 (57)	26–88%	1 (4)	0–22%	8 (38)	8–68%	10 (48)	17–79%	4 (19)	0–44%	7 (33)	3–63%

The second procedures had a higher risk of failure in comparison with first procedures, although these differences were not statistically significant (Table 15).

**Table 15:** Final IOP outcome and visual acuity according to the number of procedures

	Final IOP outcome						Visual acuity					
	Complete success		Partial success		Failure		Improvement		Unchanged		Worsening	
	*n* (%)	IC (99.61%)	*n* (%)	IC (99.61%)	*n* (%)	IC (99.61%)	*n* (%)	IC (99.61%)	*n* (%)	IC (99.61%)	*n* (%)	IC (99.61%)
First procedure	11 (52)	21–83%	2 (10)	0–28%	8 (38)	8–68%	11 (52)	21–83%	5 (24)	0–51%	5 (24)	0–51%
Second procedure	2 (33)	0–88%	1 (17)	0–61%	3 (50)	0–100%	4 (67)	12–100%	0 (0)	–	2 (33)	0–88%

Post-surgical complications included 1case each of macular edema, epiretinal membrane, endothelial injury, papillary and macular haemorrhage, subluxation of the intraocular lens and retinal detachment. There were two cases each of hyphema and diffuse bleb. There were four cases each of cystic bleb, encapsulated bleb, and plane bleb. Finally there were six cases of avascular bleb. Some cases had >1 complication and seven had no complications.

## Discussion

Estimates of the prevalence of various types of uveitis are highly variable, depending on if the data are based on cases seen at tertiary referral centres or at community-based centres. The geographic location of the study also produces different results, depending on numerous factors. The estimated prevalence in the Spanish population for each type of uveitis by anatomic classification is around 50.2%, 10.1%, 29.6% and 10.1% for anterior, intermediate, posterior uveitis and panuveitis, respectively [7].

Although some authors report no differences in the incidence of uveitic glaucoma in relation to anatomic classification and aetiology [8], our results agree with others that established anterior uveitis as the main cause of uveitic glaucoma, although only 5% of anterior uveitis patients are affected [9]. The majority of cases in our study (61.90%) were due to anterior uveitis, which is consistent with the overall distribution of uveitis. Twenty-four per cent (23.81%) of our cases were due to intermediate uveitis, about twice the prevalence in the uveitis population. This suggests that intermediate uveitis could be a high risk factor for the development of glaucoma and the consequent need for surgery. There were no cases of posterior uveitis in this study, although its prevalence in uveitis population is around 10.1%. Thus there is only a low likelihood of developing secondary uveitic glaucoma as a result of posterior uveitis. This is probably because the aqueous outflow pathway is less influenced by posterior segment inflammation, and the prevalence of IOP elevation is lower in posterior uveitis. For panuveitis, the percentage of cases developing uveitic glaucoma in our study was similar to the prevalence of uveitis in the population.

The main causes of uveitic glaucoma are juvenile chronic arthritis [10], Fuchs’ cyclitis [11, 12], Posner–Schlossman syndrome [2] and herpetic keratouveitis [13]. However, we found that the greatest risk factor for glaucoma surgery was associated with herpes, Fuchs’ cyclitis and ankylosing spondylitis. The differences between our findings and those reported by others could be that the main causes of uveitic glaucoma are not the ones that usually lead to glaucoma surgery.

Anterior idiopathic uveitis and Fuchs’ cyclitis were associated with a higher risk for failure of surgery. The absence of a satisfactory treatment for anterior idiopathic uveitis makes it difficult to manage. This in turn makes the medical treatment of glaucoma more difficult and increases the probability of failure of surgery. A portion of the patients with Fuchs’ cyclitis remains asymptomatic for a longer period than with other forms of uveitis. This may result in a relative underdiagnosis of the syndrome and perhaps an overestimate of the true frequency of secondary glaucoma resulting from it. In any case, when Fuchs’ cyclitis is complicated by glaucoma, it has a higher risk of requiring surgery and a high risk of failure.

A previous report showed that 26% of the eyes with chronic uveitis developed secondary glaucoma, compared with only 12% of the eyes with acute uveitis [14]. However, we found that most of the surgeries were associated with acute, recurrent uveitis. The increased prevalence of glaucoma in chronic uveitis reflects the accumulated detrimental effects of inflammation and probably the consequence of chronic corticosteroid therapy on an initially normal trabecular meshwork [15, 16]. However the higher rate of secondary glaucoma was not accompanied by a higher rate of glaucoma surgery [13] in contrast with our study where acute-recurrent uveitis was the main cause of glaucoma surgery.

The results were worse when surgery was necessitated prior to control of the intraocular inflammatory disease. Good control of intraocular inflammation for a minimum of 3 months before surgery is ideal but may not be practical because glaucoma surgery in uveitis is rarely performed on an elective basis. Similarly the relapse of the uveitis after the surgery was associated with a higher percentage of failure. So the control of the inflammatory process is crucial not only before of surgery but also after it to achieve a success.

Trabeculectomy has traditionally been the surgical procedure of choice for managing medically uncontrolled IOP in patients with uveitis. Previous reports have demonstrated a significant increase in the long-term success of trabeculectomy with anti-metabolic agents such as intraoperative application of MMC and intraoperative and post-operative 5-fluorouracil [17–20]. Our results with MMC are consistent with these reports.

The success rate for combined phacoemulsification and trabeculectomy approaches that of trabeculectomy alone [21]. However, there are studies reporting that trabeculectomy alone is better than the combined surgeries [22]. In our study, we found worse results with the combined surgery than with trabeculectomy alone, which seems to cause less post-operative flare than cataract surgery in isolation [23], possibly explaining the better success of trabeculectomy alone in primary open-angle glaucoma than the combined surgery [24]. It is likely that this effect is exaggerated in uveitis, and the combined cataract and glaucoma surgery may not be appropriate. Although some studies reported good outcomes combined phacoemulsification approaches in uveitic glaucoma [25], however, there are no prospective studies which compare the success rate for combined phacoemulsification and trabeculectomy approaches in uveitic glaucoma, so further studies are warranted.

Repeat surgery is less successful at achieving IOP reduction in open-angle glaucoma than is initial surgery at 3 years or more [26]. This is consistent with the results of this study in the case of uveitic glaucoma, where also a re-surgery was associated with a higher risk of failure.

In other series of surgery in uveitic glaucoma the rates of failure of the surgery in uveitic glaucoma are comparable, with a higher incidence of failure associated with longer follow-up [25, 27, 28]. Although the comparison becomes difficult because of the variability among studies to define success and the fact that in this study a lot of factors were taking into account, so further prospective and controlled studies are warranted in order to define the correct management of uveitic glaucoma. It has been reported a good outcome in uveitic glaucoma managed with trabeculectomy [25, 27, 28] and also with Ahmed valve [29, 30], and although in this study it was not found differences between both approaches, there is not studies which compare trabeculectomy and Ahmed valve in the case of uveitic glaucoma. The new aproaches such as deep sclerectomy have been also applied in the case of uveitic glaucoma without significative differences with traditional trabeculectomy, although a longer follow-up would be warranted specially in the case of uveitic glaucoma [31].

This study has the limitations of a retrospective study. Further analysis in prospective studies will help to decide the correct management for glaucoma that is secondary to the different types of uveitis. Also studies with higher population should be performed in order to increase the statistic power.
